# Methyl 5′′-chloro-1′,1′′-dimethyl-2,2′′-dioxodi­spiro­[indoline-3,2′-pyrrolidine-3′,3′′-indoline]-4′-carboxyl­ate

**DOI:** 10.1107/S1600536813011501

**Published:** 2013-05-04

**Authors:** Piskala Subburaman Kannan, PanneerSelvam Yuvaraj, Karthikeyan Manivannan, Boreddy Siva Rami Reddy, Arunachalathevar SubbiahPandi

**Affiliations:** a Department of Physics, S.M.K. Fomra Institute of Technology, Thaiyur, Chennai 603 103, India; b Industrial Chemistry Laboratory, Central Leather Research Institute, Adyar, Chennai 600 020, India; c Department of Physics, Presidency College (Autonomous), Chennai 600 005, India

## Abstract

In the title compound, C_22_H_20_ClN_3_O_4_, the central pyrrolidine ring adopts an envelope conformation on the N atom. The indolinone systems are individually roughly planar, with maximum deviations from their mean planes of 0.130 Å for the spiro C atom of the indolinone unit and 0.172 Å for the carbonyl C atom of the 5-chloro-1-methyl­indolinone unit. They make dihedral angles of 77.7 (8) and 86.1 (8)° with the mean plane through the central pyrrolidine ring. In the crystal, mol­ecules are linked by N—H⋯O hydrogen bonds supported by C—H⋯O contacts into chains along the *ab* diagonal. The structure also features C—H⋯O hydrogen bonds, forming *R*_2_^2^(8) and *R*_2_^2^(16) rings and generating a three-dimensional array.

## Related literature

For the biological activity of spiro-pyrrolidine derivatives, see: Obniska *et al.* (2003 ▶); Peddi *et al.* (2004 ▶); Kaminski & Obniska (2008 ▶); Stylianakis *et al.* (2003 ▶); Waldmann (1995 ▶). For the use of optically active pyrrolidines as inter­mediates, chiral ligands or auxiliaries in controlled asymmetric synthesis, see: Suzuki *et al.* (1994 ▶); Huryn *et al.* (1991 ▶). For related structures, see: Ganesh *et al.* (2012 ▶); Wei *et al.* (2011 ▶). For puckering parameters, see: Cremer & Pople (1975 ▶) and for hydrogen-bond motifs see Bernstein *et al.* (1995 ▶).
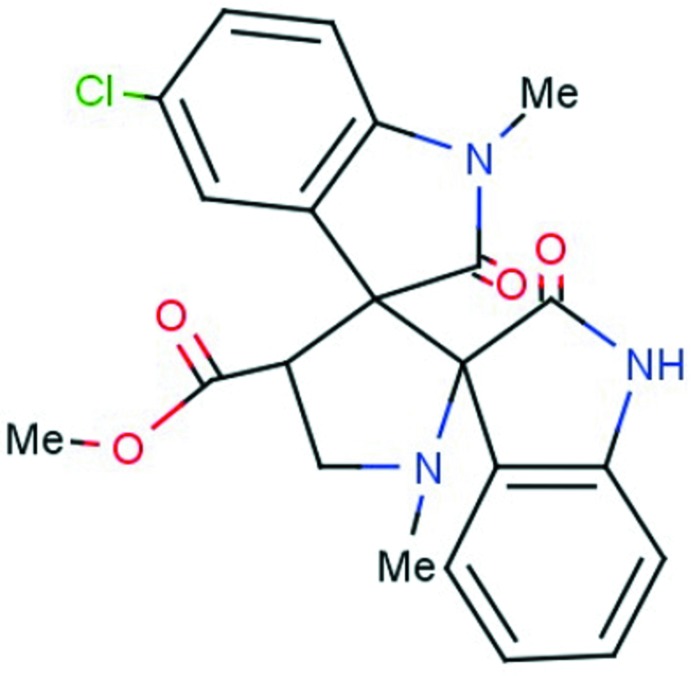


## Experimental

### 

#### Crystal data

C_22_H_20_ClN_3_O_4_
*M* *_r_* = 425.86
Monoclinic, 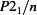

*a* = 9.2543 (4) Å
*b* = 18.1387 (7) Å
*c* = 12.5147 (5) Å
β = 105.026 (2)°
*V* = 2028.90 (14) Å^3^
*Z* = 4
Mo *K*α radiation
μ = 0.22 mm^−1^
*T* = 293 K
0.30 × 0.25 × 0.20 mm

#### Data collection

Bruker APEXII CCD area detector diffractometer
Absorption correction: multi-scan (*SADABS*; Bruker, 2008 ▶) *T*_min_ = 0.936, *T*_max_ = 0.957
18586 measured reflections
5021 independent reflections
3789 reflections with *I* > 2σ(*I*)
*R*_int_ = 0.030

#### Refinement

*R*[*F* ^2^ > 2σ(*F* ^2^)] = 0.041
*wR*(*F* ^2^) = 0.125
*S* = 1.06
5021 reflections
278 parameters
H atoms treated by a mixture of independent and constrained refinement
Δρ_max_ = 0.33 e Å^−3^
Δρ_min_ = −0.21 e Å^−3^

### 

Data collection: *APEX2* (Bruker, 2008 ▶); cell refinement: *SAINT* (Bruker, 2008 ▶); data reduction: *SAINT*; program(s) used to solve structure: *SHELXS97* (Sheldrick, 2008 ▶); program(s) used to refine structure: *SHELXL97* (Sheldrick, 2008 ▶); molecular graphics: *ORTEP-3 for Windows* (Farrugia, 2012 ▶); software used to prepare material for publication: *SHELXL97* and *PLATON* (Spek, 2009 ▶).

## Supplementary Material

Crystal structure: contains datablock(s) global, I. DOI: 10.1107/S1600536813011501/sj5312sup1.cif

Structure factors: contains datablock(s) I. DOI: 10.1107/S1600536813011501/sj5312Isup2.hkl

Additional supplementary materials:  crystallographic information; 3D view; checkCIF report

## Figures and Tables

**Table 1:** Hydrogen-bond geometry (Å, °)

*D*—H⋯*A*	*D*—H	H⋯*A*	*D*⋯*A*	*D*—H⋯*A*
N3—H3⋯O1^i^	0.85 (2)	2.18 (2)	2.9112 (18)	143.3 (18)
C15—H15⋯O1^i^	0.93	2.46	3.156 (2)	132
C22—H22*B*⋯O3^ii^	0.96	2.56	3.324 (2)	137
C5—H5⋯O3^ii^	0.93	2.61	3.499 (2)	160
C9—H9⋯O3^iii^	0.98	2.54	3.224 (2)	127

Symmetry codes: (i) 
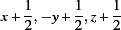
; (ii) 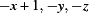; (iii) 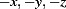.

**Fig. 1.**
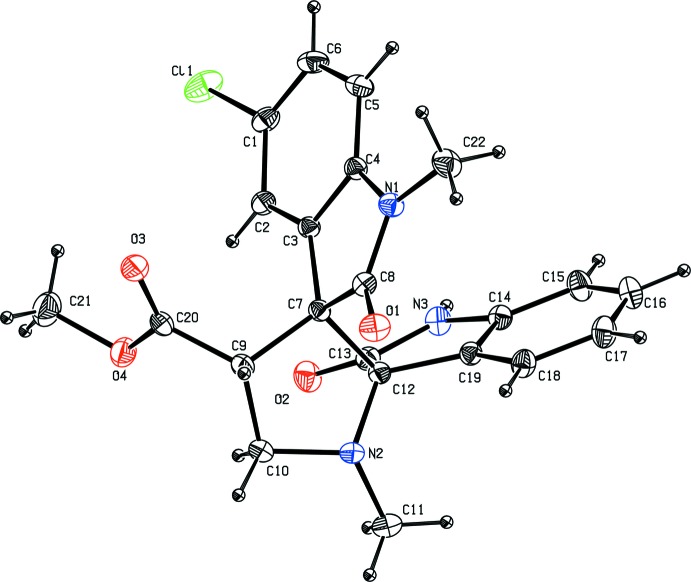
The structure of showing the atom-numbering scheme. The displacement ellipsoids are drawn at the 30% probability level.

**Fig. 2.**
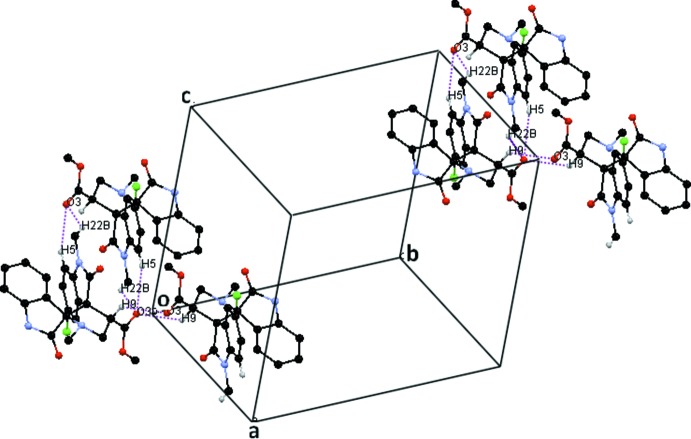
The molecular packing viewed along a. Dashed lines show the intermolecular N–H···O and C–H···O hydrogen bonds.

## supplementary crystallographic information

### Comment

Spiro-pyrrolidine derivatives are unique tetracyclic 5-HT(2A) receptor antagonists (Obniska *et al.*, 2003; Peddi *et al.*, 2004). These derivatives possess anticonvulsant (Kaminski & Obniska, 2008) and anti-influenza virus (Stylianakis *et al.*, 2003) activities. Highly functionalized pyrrolidines have gained much interest in the past few years as they constitute the main structural element of many natural and synthetic pharmacologically active compounds (Waldmann, 1995). Optically active pyrrolidines have also been used as intermediates, chiral ligands or auxiliaries in controlled asymmetric synthesis (Suzuki *et al.*, 1994; Huryn *et al.*, 1991).

X-Ray analysis confirms the molecular structure and atom connectivity as illustrated in Fig. 1. The geometries of the pyrrolidine and indole systems are comparable with those in related structures (Wei *et al.*, 2011; Ganesh *et al.*, 2012). The sum of the angles at N2 [336.9 (1)°] of the pyrrolidine rings is typical of *sp*^3^ hybridization. The indoline ring systems [N1/C1-C8 and N3/C12-C19] make dihedral angles of 77.7 (8) ° and 86.1(68° with respect to the mean plane of the central pyrrolidine ring system [N2/C7/C9/C10/C12]. This clearly shows that the indoline ring (N3/C12-C19) and the central pyrrolidine ring system are almost perpendicular to one another. The indole ring systems are essentially planar, with maximum deviations from the mean planes of 0.130 Å for the C12 and -0.172 Å for the C8 atoms, respectively.

The central pyrrolidine ring adopts an envelope conformation on the N2 atom, with puckering parameters q_2_ = 0.419 (2) Å, φ = 1.555 (2)(Cremer & Pople, 1975 ). The pyrrolidine ring in the chloro-indole ring system adopts a twisted conformation on the C7 and C8 atoms, with puckering parameters of q_2_ = 0.124 (2) Å, φ = 306.8 (7). The pyrrolidine ring in the indole ring system adopts an envelope conformation on the C12 atom, with puckering parameters q_2_ = 0.113 (2) Å, φ = 251.6 (9).

In the crystal, six hydrogen bonds formed by each molecule. These include the formation of three inversion related contacts and atom O3 acting as a trifurcated acceptor. The molecules are stabilized by intermolecular C–H···O hydrogen bonds forming R^2^_2_(8)rings from C9-H9···O3, contacts (Bernstein *et al.*, 1995) while C5–H5···O3 contacts and C22-H22B···O3, H bonds generate R^2^_2_(16) rings resulting in a three dimensional array Figure 2.

### Experimental

A mixture of 1 equivalent of (*E*)-methyl 2-(5-chloro-1-methyl-2-oxoindolin-3-ylidene) acetate, 1 equivalent of isatin, 1H-indole-2,3-dione, and 1.5 equivalent of sarcosine, *N*-methylglycine were dissolved in acetonitrile. This reaction mixture was refluxed at 80°C for 8 hours. Progress of the reaction was monitored by thin layer chromatography. The product was dried and purified by column chromatography using ethyl acetate and hexane (1:9) as eluent to afford the title compound. (Yield = 90%). Single crystals suitable for X-ray diffraction were obtained by slow evaporation of a solution in ethyl acetate at room temperature.

### Refinement

The H atom bound to N3 was located in a difference Fourier map and its coordinates and atomic displacement parameter were refined freely. All H atoms bound to C were fixed geometrically and allowed to ride on their parent atoms, with C—H distances fixed in the range 0.93–0.97 Å and with *U*_iso_(H) = 1.5*U*_eq_(C) for methyl H 1.2*U*_eq_(C) for other H atoms. The positions of methyl hydrogens were optimized rotationally.

### Crystal data

**Table:**

C_22_H_20_ClN_3_O_4_	*F*(000) = 888
*M**_r_* = 425.86	*D*_x_ = 1.394 Mg m^−^^3^
Monoclinic, *P*2_1_/*n*	Mo *K*α radiation, λ = 0.71073 Å
Hall symbol: -P 2yn	Cell parameters from 5021 reflections
*a* = 9.2543 (4) Å	θ = 2.0–28.3°
*b* = 18.1387 (7) Å	µ = 0.22 mm^−^^1^
*c* = 12.5147 (5) Å	*T* = 293 K
β = 105.026 (2)°	Block, colourless
*V* = 2028.90 (14) Å^3^	0.30 × 0.25 × 0.20 mm
*Z* = 4	

### Data collection

**Table:**

Bruker APEXII CCD area detector diffractometer	5021 independent reflections
Radiation source: fine-focus sealed tube	3789 reflections with *I* > 2σ(*I*)
Graphite monochromator	*R*_int_ = 0.030
ω and φ scans	θ_max_ = 28.3°, θ_min_ = 2.0°
Absorption correction: multi-scan (*SADABS*; Bruker, 2008)	*h* = −12→12
*T*_min_ = 0.936, *T*_max_ = 0.957	*k* = −24→24
18586 measured reflections	*l* = −16→16

### Refinement

**Table:**

Refinement on *F*^2^	Primary atom site location: structure-invariant direct methods
Least-squares matrix: full	Secondary atom site location: difference Fourier map
*R*[*F*^2^ > 2σ(*F*^2^)] = 0.041	Hydrogen site location: inferred from neighbouring sites
*wR*(*F*^2^) = 0.125	H atoms treated by a mixture of independent and constrained refinement
*S* = 1.06	*w* = 1/[σ^2^(*F*_o_^2^) + (0.0572*P*)^2^ + 0.6292*P*] where *P* = (*F*_o_^2^ + 2*F*_c_^2^)/3
5021 reflections	(Δ/σ)_max_ < 0.001
278 parameters	Δρ_max_ = 0.33 e Å^−^^3^
0 restraints	Δρ_min_ = −0.21 e Å^−^^3^

### Special details

**Table:**

Geometry. All esds (except the esd in the dihedral angle between two l.s. planes) are estimated using the full covariance matrix. The cell esds are taken into account individually in the estimation of esds in distances, angles and torsion angles; correlations between esds in cell parameters are only used when they are defined by crystal symmetry. An approximate (isotropic) treatment of cell esds is used for estimating esds involving l.s. planes.
Refinement. Refinement of F^2^ against ALL reflections. The weighted R-factor wR and goodness of fit S are based on F^2^, conventional R-factors R are based on F, with F set to zero for negative F^2^. The threshold expression of F^2^ > 2sigma(F^2^) is used only for calculating R-factors(gt) etc. and is not relevant to the choice of reflections for refinement. R-factors based on F^2^ are statistically about twice as large as those based on F, and R- factors based on ALL data will be even larger.

### Fractional atomic coordinates and isotropic or equivalent isotropic displacement parameters (Å^2^)

**Table:**

	*x*	*y*	*z*	*U*_iso_*/*U*_eq_	
C1	0.59371 (19)	0.05207 (9)	0.28439 (13)	0.0423 (4)	
C2	0.45435 (17)	0.08424 (8)	0.24338 (12)	0.0358 (3)	
H2	0.3891	0.0908	0.2880	0.043*	
C3	0.41592 (15)	0.10629 (8)	0.13379 (12)	0.0301 (3)	
C4	0.51723 (16)	0.09636 (8)	0.06936 (12)	0.0334 (3)	
C5	0.65753 (18)	0.06619 (10)	0.11172 (15)	0.0446 (4)	
H5	0.7247	0.0612	0.0683	0.053*	
C6	0.69446 (19)	0.04367 (10)	0.22136 (16)	0.0486 (4)	
H6	0.7877	0.0228	0.2525	0.058*	
C7	0.27545 (15)	0.14084 (8)	0.06175 (11)	0.0294 (3)	
C8	0.30532 (17)	0.13446 (8)	−0.05324 (12)	0.0340 (3)	
C9	0.11746 (16)	0.10891 (9)	0.05792 (13)	0.0365 (3)	
H9	0.0726	0.0954	−0.0194	0.044*	
C10	0.02656 (18)	0.17386 (9)	0.08235 (17)	0.0473 (4)	
H10A	−0.0777	0.1698	0.0417	0.057*	
H10B	0.0331	0.1775	0.1608	0.057*	
C11	0.0415 (2)	0.30826 (11)	0.0677 (2)	0.0601 (5)	
H11A	0.0551	0.3134	0.1460	0.090*	
H11B	−0.0631	0.3117	0.0308	0.090*	
H11C	0.0949	0.3467	0.0417	0.090*	
C12	0.25861 (16)	0.22640 (8)	0.08568 (12)	0.0319 (3)	
C13	0.32532 (18)	0.24031 (8)	0.21214 (13)	0.0361 (3)	
C14	0.47566 (18)	0.30011 (8)	0.12068 (13)	0.0362 (3)	
C15	0.5915 (2)	0.34053 (11)	0.09938 (15)	0.0489 (4)	
H15	0.6722	0.3557	0.1563	0.059*	
C16	0.5830 (2)	0.35771 (11)	−0.00992 (16)	0.0536 (5)	
H16	0.6609	0.3837	−0.0267	0.064*	
C17	0.4617 (2)	0.33718 (11)	−0.09432 (15)	0.0507 (4)	
H17	0.4572	0.3508	−0.1668	0.061*	
C18	0.3457 (2)	0.29619 (9)	−0.07182 (14)	0.0426 (4)	
H18	0.2630	0.2828	−0.1284	0.051*	
C19	0.35598 (16)	0.27576 (8)	0.03628 (12)	0.0333 (3)	
C20	0.12538 (16)	0.03924 (9)	0.12354 (14)	0.0397 (4)	
C21	0.1033 (3)	−0.01945 (12)	0.2852 (2)	0.0683 (6)	
H21A	0.0331	−0.0556	0.2467	0.102*	
H21B	0.0828	−0.0082	0.3548	0.102*	
H21C	0.2030	−0.0385	0.2980	0.102*	
C22	0.5254 (2)	0.11715 (12)	−0.12913 (15)	0.0528 (5)	
H22A	0.4533	0.1265	−0.1982	0.079*	
H22B	0.5717	0.0701	−0.1322	0.079*	
H22C	0.6004	0.1550	−0.1158	0.079*	
N1	0.45105 (15)	0.11694 (7)	−0.04029 (10)	0.0367 (3)	
N2	0.09833 (14)	0.23692 (7)	0.04428 (12)	0.0406 (3)	
N3	0.45486 (16)	0.27829 (8)	0.22362 (12)	0.0406 (3)	
O1	0.21387 (13)	0.14407 (7)	−0.14168 (9)	0.0467 (3)	
O2	0.27254 (15)	0.22036 (7)	0.28633 (10)	0.0516 (3)	
O3	0.16251 (15)	−0.01881 (7)	0.09239 (12)	0.0535 (3)	
O4	0.08942 (15)	0.04712 (7)	0.21895 (11)	0.0513 (3)	
Cl1	0.63981 (7)	0.01829 (3)	0.41915 (4)	0.07053 (19)	
H3	0.513 (2)	0.2916 (11)	0.2850 (18)	0.049 (5)*	

### Atomic displacement parameters (Å^2^)

**Table:**

	*U*^11^	*U*^22^	*U*^33^	*U*^12^	*U*^13^	*U*^23^
C1	0.0456 (9)	0.0388 (8)	0.0358 (8)	0.0052 (7)	−0.0016 (7)	0.0025 (6)
C2	0.0376 (8)	0.0349 (7)	0.0336 (8)	0.0014 (6)	0.0071 (6)	0.0003 (6)
C3	0.0270 (6)	0.0294 (7)	0.0325 (7)	−0.0002 (5)	0.0053 (5)	−0.0014 (5)
C4	0.0305 (7)	0.0335 (7)	0.0356 (8)	−0.0014 (6)	0.0076 (6)	−0.0012 (6)
C5	0.0316 (8)	0.0501 (9)	0.0524 (10)	0.0058 (7)	0.0117 (7)	−0.0023 (8)
C6	0.0341 (8)	0.0506 (10)	0.0540 (10)	0.0111 (7)	−0.0014 (7)	−0.0009 (8)
C7	0.0263 (7)	0.0323 (7)	0.0286 (7)	−0.0005 (5)	0.0052 (5)	−0.0001 (5)
C8	0.0367 (8)	0.0330 (7)	0.0310 (7)	−0.0001 (6)	0.0067 (6)	−0.0023 (6)
C9	0.0269 (7)	0.0396 (8)	0.0411 (8)	−0.0036 (6)	0.0054 (6)	0.0015 (6)
C10	0.0304 (8)	0.0446 (9)	0.0689 (12)	0.0030 (7)	0.0162 (8)	0.0085 (8)
C11	0.0521 (11)	0.0455 (10)	0.0865 (15)	0.0154 (8)	0.0250 (10)	0.0088 (10)
C12	0.0308 (7)	0.0327 (7)	0.0323 (7)	0.0014 (6)	0.0082 (6)	0.0022 (6)
C13	0.0410 (8)	0.0346 (7)	0.0343 (8)	0.0038 (6)	0.0130 (6)	−0.0011 (6)
C14	0.0392 (8)	0.0342 (7)	0.0354 (8)	−0.0014 (6)	0.0099 (6)	−0.0026 (6)
C15	0.0441 (9)	0.0532 (10)	0.0481 (10)	−0.0141 (8)	0.0093 (8)	−0.0031 (8)
C16	0.0530 (11)	0.0557 (11)	0.0569 (11)	−0.0133 (8)	0.0231 (9)	0.0038 (9)
C17	0.0635 (11)	0.0524 (10)	0.0403 (9)	−0.0060 (9)	0.0206 (8)	0.0075 (8)
C18	0.0465 (9)	0.0432 (9)	0.0353 (8)	−0.0043 (7)	0.0057 (7)	0.0043 (7)
C19	0.0350 (7)	0.0313 (7)	0.0340 (7)	−0.0006 (6)	0.0100 (6)	−0.0004 (6)
C20	0.0264 (7)	0.0416 (8)	0.0490 (9)	−0.0062 (6)	0.0059 (6)	−0.0003 (7)
C21	0.0829 (16)	0.0602 (13)	0.0651 (14)	0.0020 (11)	0.0252 (12)	0.0181 (10)
C22	0.0556 (11)	0.0664 (12)	0.0441 (10)	0.0031 (9)	0.0265 (8)	0.0001 (8)
N1	0.0373 (7)	0.0425 (7)	0.0319 (6)	0.0029 (5)	0.0122 (5)	−0.0009 (5)
N2	0.0295 (6)	0.0379 (7)	0.0538 (8)	0.0059 (5)	0.0096 (6)	0.0070 (6)
N3	0.0455 (8)	0.0442 (8)	0.0300 (7)	−0.0070 (6)	0.0060 (6)	−0.0053 (6)
O1	0.0484 (7)	0.0567 (7)	0.0291 (6)	0.0048 (6)	−0.0004 (5)	−0.0010 (5)
O2	0.0606 (8)	0.0612 (8)	0.0394 (6)	−0.0015 (6)	0.0243 (6)	0.0023 (6)
O3	0.0497 (7)	0.0394 (7)	0.0746 (9)	0.0012 (5)	0.0218 (7)	−0.0042 (6)
O4	0.0610 (8)	0.0437 (7)	0.0529 (7)	0.0004 (6)	0.0213 (6)	0.0059 (6)
Cl1	0.0869 (4)	0.0727 (4)	0.0425 (3)	0.0259 (3)	−0.0003 (2)	0.0164 (2)

### Geometric parameters (Å, º)

**Table:**

C1—C6	1.377 (3)	C12—N2	1.4514 (18)
C1—C2	1.387 (2)	C12—C19	1.511 (2)
C1—Cl1	1.7403 (17)	C12—C13	1.563 (2)
C2—C3	1.384 (2)	C13—O2	1.2110 (19)
C2—H2	0.9300	C13—N3	1.358 (2)
C3—C4	1.398 (2)	C14—C15	1.381 (2)
C3—C7	1.5125 (19)	C14—C19	1.390 (2)
C4—C5	1.382 (2)	C14—N3	1.408 (2)
C4—N1	1.401 (2)	C15—C16	1.385 (3)
C5—C6	1.387 (3)	C15—H15	0.9300
C5—H5	0.9300	C16—C17	1.378 (3)
C6—H6	0.9300	C16—H16	0.9300
C7—C8	1.539 (2)	C17—C18	1.393 (2)
C7—C9	1.5617 (19)	C17—H17	0.9300
C7—C12	1.596 (2)	C18—C19	1.382 (2)
C8—O1	1.2190 (18)	C18—H18	0.9300
C8—N1	1.354 (2)	C20—O3	1.203 (2)
C9—C20	1.499 (2)	C20—O4	1.328 (2)
C9—C10	1.524 (2)	C21—O4	1.452 (2)
C9—H9	0.9800	C21—H21A	0.9600
C10—N2	1.463 (2)	C21—H21B	0.9600
C10—H10A	0.9700	C21—H21C	0.9600
C10—H10B	0.9700	C22—N1	1.451 (2)
C11—N2	1.455 (2)	C22—H22A	0.9600
C11—H11A	0.9600	C22—H22B	0.9600
C11—H11B	0.9600	C22—H22C	0.9600
C11—H11C	0.9600	N3—H3	0.85 (2)
			
C6—C1—C2	122.50 (15)	C19—C12—C7	113.69 (12)
C6—C1—Cl1	118.94 (13)	C13—C12—C7	108.35 (11)
C2—C1—Cl1	118.52 (14)	O2—C13—N3	126.24 (15)
C3—C2—C1	117.63 (15)	O2—C13—C12	126.59 (15)
C3—C2—H2	121.2	N3—C13—C12	107.17 (13)
C1—C2—H2	121.2	C15—C14—C19	121.86 (15)
C2—C3—C4	119.74 (13)	C15—C14—N3	128.56 (15)
C2—C3—C7	132.13 (13)	C19—C14—N3	109.57 (13)
C4—C3—C7	108.12 (12)	C14—C15—C16	117.50 (16)
C5—C4—C3	122.27 (14)	C14—C15—H15	121.2
C5—C4—N1	127.60 (14)	C16—C15—H15	121.2
C3—C4—N1	110.03 (13)	C17—C16—C15	121.52 (17)
C4—C5—C6	117.53 (15)	C17—C16—H16	119.2
C4—C5—H5	121.2	C15—C16—H16	119.2
C6—C5—H5	121.2	C16—C17—C18	120.42 (16)
C1—C6—C5	120.29 (15)	C16—C17—H17	119.8
C1—C6—H6	119.9	C18—C17—H17	119.8
C5—C6—H6	119.9	C19—C18—C17	118.67 (16)
C3—C7—C8	101.03 (11)	C19—C18—H18	120.7
C3—C7—C9	121.19 (12)	C17—C18—H18	120.7
C8—C7—C9	109.70 (12)	C18—C19—C14	119.87 (14)
C3—C7—C12	113.69 (11)	C18—C19—C12	131.46 (14)
C8—C7—C12	107.37 (11)	C14—C19—C12	108.64 (13)
C9—C7—C12	103.31 (11)	O3—C20—O4	123.00 (16)
O1—C8—N1	125.30 (14)	O3—C20—C9	122.54 (16)
O1—C8—C7	126.00 (14)	O4—C20—C9	114.46 (14)
N1—C8—C7	108.70 (12)	O4—C21—H21A	109.5
C20—C9—C10	119.54 (14)	O4—C21—H21B	109.5
C20—C9—C7	112.55 (12)	H21A—C21—H21B	109.5
C10—C9—C7	105.56 (12)	O4—C21—H21C	109.5
C20—C9—H9	106.1	H21A—C21—H21C	109.5
C10—C9—H9	106.1	H21B—C21—H21C	109.5
C7—C9—H9	106.1	N1—C22—H22A	109.5
N2—C10—C9	102.60 (13)	N1—C22—H22B	109.5
N2—C10—H10A	111.2	H22A—C22—H22B	109.5
C9—C10—H10A	111.2	N1—C22—H22C	109.5
N2—C10—H10B	111.2	H22A—C22—H22C	109.5
C9—C10—H10B	111.2	H22B—C22—H22C	109.5
H10A—C10—H10B	109.2	C8—N1—C4	110.42 (12)
N2—C11—H11A	109.5	C8—N1—C22	124.27 (14)
N2—C11—H11B	109.5	C4—N1—C22	125.28 (14)
H11A—C11—H11B	109.5	C12—N2—C11	115.75 (14)
N2—C11—H11C	109.5	C12—N2—C10	106.81 (12)
H11A—C11—H11C	109.5	C11—N2—C10	114.29 (14)
H11B—C11—H11C	109.5	C13—N3—C14	111.86 (14)
N2—C12—C19	116.02 (12)	C13—N3—H3	125.2 (13)
N2—C12—C13	116.05 (12)	C14—N3—H3	122.7 (13)
C19—C12—C13	101.40 (12)	C20—O4—C21	114.76 (15)
N2—C12—C7	101.61 (11)		
			
C6—C1—C2—C3	2.0 (2)	C19—C12—C13—N3	−11.00 (15)
Cl1—C1—C2—C3	−175.78 (11)	C7—C12—C13—N3	108.91 (13)
C1—C2—C3—C4	−0.6 (2)	C19—C14—C15—C16	1.4 (3)
C1—C2—C3—C7	178.34 (15)	N3—C14—C15—C16	−177.23 (17)
C2—C3—C4—C5	−1.2 (2)	C14—C15—C16—C17	1.8 (3)
C7—C3—C4—C5	179.57 (14)	C15—C16—C17—C18	−2.1 (3)
C2—C3—C4—N1	175.27 (13)	C16—C17—C18—C19	−0.9 (3)
C7—C3—C4—N1	−3.92 (16)	C17—C18—C19—C14	4.1 (2)
C3—C4—C5—C6	1.8 (2)	C17—C18—C19—C12	−173.57 (16)
N1—C4—C5—C6	−174.09 (15)	C15—C14—C19—C18	−4.4 (2)
C2—C1—C6—C5	−1.4 (3)	N3—C14—C19—C18	174.48 (14)
Cl1—C1—C6—C5	176.30 (14)	C15—C14—C19—C12	173.71 (16)
C4—C5—C6—C1	−0.5 (3)	N3—C14—C19—C12	−7.39 (17)
C2—C3—C7—C8	−169.10 (15)	N2—C12—C19—C18	−44.6 (2)
C4—C3—C7—C8	9.95 (15)	C13—C12—C19—C18	−171.21 (16)
C2—C3—C7—C9	−47.8 (2)	C7—C12—C19—C18	72.7 (2)
C4—C3—C7—C9	131.25 (14)	N2—C12—C19—C14	137.60 (14)
C2—C3—C7—C12	76.21 (19)	C13—C12—C19—C14	10.95 (15)
C4—C3—C7—C12	−104.73 (13)	C7—C12—C19—C14	−105.10 (14)
C3—C7—C8—O1	167.44 (15)	C10—C9—C20—O3	161.37 (16)
C9—C7—C8—O1	38.4 (2)	C7—C9—C20—O3	−73.93 (19)
C12—C7—C8—O1	−73.23 (18)	C10—C9—C20—O4	−19.2 (2)
C3—C7—C8—N1	−13.09 (15)	C7—C9—C20—O4	105.49 (15)
C9—C7—C8—N1	−142.15 (13)	O1—C8—N1—C4	−168.84 (15)
C12—C7—C8—N1	106.24 (13)	C7—C8—N1—C4	11.68 (16)
C3—C7—C9—C20	−5.1 (2)	O1—C8—N1—C22	9.1 (3)
C8—C7—C9—C20	111.93 (14)	C7—C8—N1—C22	−170.33 (15)
C12—C7—C9—C20	−133.82 (13)	C5—C4—N1—C8	171.24 (16)
C3—C7—C9—C10	126.97 (15)	C3—C4—N1—C8	−5.04 (17)
C8—C7—C9—C10	−116.01 (14)	C5—C4—N1—C22	−6.7 (3)
C12—C7—C9—C10	−1.77 (15)	C3—C4—N1—C22	177.01 (15)
C20—C9—C10—N2	154.82 (14)	C19—C12—N2—C11	−64.38 (19)
C7—C9—C10—N2	26.84 (17)	C13—C12—N2—C11	54.53 (19)
C3—C7—C12—N2	−157.26 (12)	C7—C12—N2—C11	171.79 (14)
C8—C7—C12—N2	91.88 (13)	C19—C12—N2—C10	167.10 (14)
C9—C7—C12—N2	−24.03 (14)	C13—C12—N2—C10	−73.99 (17)
C3—C7—C12—C19	77.35 (15)	C7—C12—N2—C10	43.27 (15)
C8—C7—C12—C19	−33.51 (15)	C9—C10—N2—C12	−45.11 (17)
C9—C7—C12—C19	−149.42 (12)	C9—C10—N2—C11	−174.47 (15)
C3—C7—C12—C13	−34.55 (16)	O2—C13—N3—C14	−173.29 (16)
C8—C7—C12—C13	−145.41 (12)	C12—C13—N3—C14	7.43 (17)
C9—C7—C12—C13	98.68 (13)	C15—C14—N3—C13	178.58 (17)
N2—C12—C13—O2	43.1 (2)	C19—C14—N3—C13	−0.23 (19)
C19—C12—C13—O2	169.72 (16)	O3—C20—O4—C21	2.0 (2)
C7—C12—C13—O2	−70.37 (19)	C9—C20—O4—C21	−177.38 (16)
N2—C12—C13—N3	−137.63 (14)		

### Hydrogen-bond geometry (Å, º)

**Table:**

*D*—H···*A*	*D*—H	H···*A*	*D*···*A*	*D*—H···*A*
N3—H3···O1^i^	0.85 (2)	2.18 (2)	2.9112 (18)	143.3 (18)
C15—H15···O1^i^	0.93	2.46	3.156 (2)	132
C22—H22*B*···O3^ii^	0.96	2.56	3.324 (2)	137
C5—H5···O3^ii^	0.93	2.61	3.499 (2)	160
C9—H9···O3^iii^	0.98	2.54	3.224 (2)	127
C9—H9···O1	0.98	2.42	2.932 (2)	112

Symmetry codes: (i) *x*+1/2, −*y*+1/2, *z*+1/2; (ii) −*x*+1, −*y*, −*z*; (iii) −*x*, −*y*, −*z*.
